# 

**Published:** 1865-07

**Authors:** 


### CHICAGO MEDICAL JOURNAL.

**Terms, $2.00 per Annum.**

### CONTENTS OF THE JULY NUMBER.

PAGE.

ORIGINAL COMMUNICATIONS.

Cerebro—Spinal Meningitis, or Spotted Fever. By Ephraim Ingals, M. D .......... 289

Gun-Shot Wound through the Heart. A Thesis for the Degree of Doctor of Medicine, presented to the Faculty of Rush Medical College, June 25th, 1865. By W. Ellery Bowman .......... 299

SELECTED.

On the Treatment of Pneumonia by Restoratives. By John Hughes Bennett, M. D .......... 304

Smoking as a Cause of Fatty Heart .......... 310

Permenganate of Potash as a Remedy for Diphtheria. By Louis Mackall, Jr., M. D., Georgetown, D. C .......... 312

Permanganate of Potash in Gonorrhœa .......... 314

Persulphate of Iron in Hemorrhoids .......... 315

Hints For the Traveling Season .......... 316

Longevity .......... 317

Epilepsy and the Administration of Bromide of Potassium. By G. Goddard Rogers, M. D., Physician to the West London Hospital, etc. 318

EDITORIAL AND MISCELLANEOUS.

An Example Worthy of Imitation .......... 323

American Medical Association—Sixteenth Annual Convention .......... 325

Proceedings of De Witt County Medical Society. Reported by C. Goodbrake, M. D., Secretary .......... 334

